# Corticosteroid suppresses urea-cycle-related gene expressions in ornithine transcarbamylase deficiency

**DOI:** 10.1186/s12876-022-02213-0

**Published:** 2022-03-28

**Authors:** Koji Imoto, Masatake Tanaka, Takeshi Goya, Tomomi Aoyagi, Motoi Takahashi, Miho Kurokawa, Shigeki Tashiro, Masaki Kato, Motoyuki Kohjima, Yoshihiro Ogawa

**Affiliations:** 1 Department of Medicine and Bioregulatory Science, Graduate School of Medical Sciences, Kyushu University, 3-1-1 Maidashi, Higashi-Ku, Fukuoka, 812-8582 Japan; 2 Graduate School of Nutritional Sciences, Nakamura Gakuen University, 5-7-1 Befu, Jounan-Ku, Fukuoka, 814-0198 Japan

**Keywords:** Ornithine transcarbamylase deficiency, Corticosteroid, Hyperammonemia, Urea cycle disorder, Late-onset ornithine transcarbamylase deficiency

## Abstract

**Background:**

Ornithine transcarbamylase deficiency (OTCD) is most common among urea cycle disorders (UCDs), defined by defects in enzymes associated with ureagenesis. Corticosteroid administration to UCD patients, including OTCD patients, is suggested to be avoided, as it may induce life-threatening hyperammonemia. The mechanism has been considered nitrogen overload due to the catabolic effect of corticosteroids; however, the pathophysiological process is unclear.

**Methods:**

To elucidate the mechanism of hyperammonemia induced by corticosteroid administration in OTCD patients, we analyzed a mouse model by administering corticosteroids to OTC^spf−ash^ mice deficient in the OTC gene. Dexamethasone (DEX; 20 mg/kg) was administered to the OTC^spf−ash^ and wild-type (WT) mice at 0 and 24 h, and the serum ammonia concentrations, the levels of the hepatic metabolites, and the gene expressions related with ammonia metabolism in the livers and muscles were analyzed.

**Results:**

The ammonia levels in Otc^spf−ash^ mice that were administered DEX tended to increase at 24 h and increased significantly at 48 h. The metabolomic analysis showed that the levels of citrulline, arginine, and ornithine did not differ significantly between Otc^spf−ash^ mice that were administered DEX and normal saline; however, the level of aspartate was increased drastically in Otc^spf−ash^ mice owing to DEX administration (*P* < 0.01). Among the enzymes associated with the urea cycle, mRNA expressions of carbamoyl-phosphate synthase 1, ornithine transcarbamylase, arginosuccinate synthase 1, and arginosuccinate lyase in the livers were significantly downregulated by DEX administration in both the Otc^spf−ash^ and WT mice (*P* < 0.01). Among the enzymes associated with catabolism, mRNA expression of Muscle RING-finger protein-1 in the muscles was significantly upregulated in the muscles of WT mice by DEX administration (*P* < 0.05).

**Conclusions:**

We elucidated that corticosteroid administration induced hyperammonemia in Otc^spf−ash^ mice by not only muscle catabolism but also suppressing urea-cycle-related gene expressions. Since the urea cycle intermediate amino acids, such as arginine, might not be effective because of the suppressed expression of urea-cycle-related genes by corticosteroid administration, we should consider an early intervention by renal replacement therapy in cases of UCD patients induced by corticosteroids to avoid brain injuries or fatal outcomes.

**Supplementary Information:**

The online version contains supplementary material available at 10.1186/s12876-022-02213-0.

## Background

UCDs are inherited metabolic diseases resulting from the complete or partial inactivity of any of the enzymes associated with the urea cycle, which is responsible for removing nitrogenous waste. The nitrogen accumulates in the form of ammonia, and unless ammonia converts to urea, increased ammonia leads to life-threatening encephalopathy [1]. UCDs have an estimated incidence of 1 in every 8000–44,000 births [2]. Ornithine transcarbamylase deficiency (OTCD) is transmitted as an X-linked trait, and it is the most common UCD; the prevalence of OTCD in Japan is 1 in 80,000 people [3]. The phenotype of OTCD is highly heterogeneous, ranging from acute neonatal hyperammonemic coma [4] to a complete absence of symptoms in hemizygous males who might become symptomatic only much later in life [5]. These phenotypic differences are associated with the degree of residual enzyme activity [5]. OTCD most frequently occurs in children up to 5 years of age; however, it occurs in patients aged > 5 years in approximately 20% of cases [3]. There have been more reports of adult-onset cases recently, and these patients may die or suffer from serious complications [5–8]. We have experienced two unexplained hyperammonemic patients with corticosteroids, and they received multimodal treatment, including dialysis. They recovered completely from severe hyperammonemia and were finally diagnosed with late-onset OTCD. A variety of causes, including dietary non-adherence, enhanced protein catabolism due to protein or caloric over-restriction, infection, gastrointestinal bleeding, and corticosteroids, caused hyperammonemia in patients with UCD [9]. Although corticosteroid-induced hyperammonemia in UCD patients is supposed to result from increased protein catabolism [7], both of our patients presented drastic exacerbation of hyperammonemia in a short period of time. Their clinical features indicated that corticosteroid-induced hyperammonemia in UCD patients could be explained not only by protein catabolism alteration but also by more rapid physiological changes. Glucocorticoids increase the gene expression levels of Arginase 1 *(ARG1)* and carbamoyl-phosphate synthase 1 *(CPS1)* in adult rat hepatocytes [10–12], but the effect of corticosteroids on the urea cycle in UCD patients is not clear.

The current paradigm for acute hyperammonemia treatment addresses the increased whole-body protein catabolism regardless of the causes [9]. However, the pathophysiological processes behind the different causes of hyperammonemia might be distinct, which raises the possibility of targeted therapies that alter the prognosis of UCD patients.

We presented two late-onset OTCD patients who received corticosteroids to understand the clinical features of hyperammonemia in OTCD patients receiving corticosteroids (Additional file 1 and Additional file 1: Fig. S1). We also undertook a translational approach to elucidate the mechanism of acute hyperammonemia in OTCD with corticosteroids using an experimental model of corticosteroid-associated acute hyperammonemia utilized by administering corticosteroids to Otc^spf−ash^ mice, a mouse model of OTCD.

## Methods

### Animals

Otc^spf−ash^ mice (Otc^spf−ash^, originally on C3H-F1 background) were purchased from the Jackson Laboratory (B6EiC3Sn a/A-Otcspf-ash/J). Twelve-week-old hemizygous Otc^spf−ash^ and wild-type (WT) males were used. All animals were acclimated to the environment in a temperature-, humidity-, and light-controlled room (12 h light and 12 h dark cycle) and were allowed access to water and a standard diet ad libitum (CE-2; 340.2 kcal/100 g, 24.8% energy as protein; CLEA Japan). Mice were treated with 20 mg/kg body wt of dexamethasone (DEX; catalog no. D 2915; Sigma, St. Louis, MO) in 0.9% normal saline by intraperitoneal injection at 0 and 24 h (Otc^spf−ash^ mice; n = 5, WT mice; n = 3). Mice were made to fast for 3 to 5 h, and blood samples were collected from the tail vein at 0, 24, and 48 h after the first DEX injection [13]. Control animals underwent sham injections with 0.9% normal saline (Otc^spf−ash^ mice; n = 5, WT mice; n = 3). The mice in the control and DEX groups were sacrificed at 48 h. All animals were euthanized by isoflurane, and the livers and gastrocnemius muscles were harvested. The livers and gastrocnemius muscles were immediately frozen in liquid nitrogen for mRNA extraction and metabolomic analysis. All studies were performed following the Guide for the Care and Use of Laboratory Animals (National Institutes of Health) and approved by the Animal Care Committee of Kyushu University.

### Biochemical analyses

Serum levels of ammonia were measured using a Fuji-Drychem chemical analyzer NX500sV (Fuji Film, Tokyo, Japan).

### Histological analysis

For histological evaluation, liver and muscle tissues were fixed in 10% buffered formalin and embedded in paraffin and Hematoxylin and Eosin (HE) staining.

### Metabolomic analysis

The metabolomic analysis was performed by Kyushu Pro Search LLP (Fukuoka, Japan). In brief, the liver samples were homogenized using beads and suspended in 700 μL of distilled water and were mixed with methanol (2 mL) and chloroform (2 mL) for 10 min at room temperature. After centrifugation at 1000×*g* for 15 min, the supernatant was evaporated using nitrogen gas and dissolved in 10% acetonitrile aqueous solution (200 μL). After adding internal standards, the samples were subjected to both liquid chromatography–mass spectrometry and capillary electrophoresis–mass spectrometry. A data file of mass spectrometry was converted to CSV format with an Agilent CSV convertor. All peak positions (retention time and m/z) and areas were calculated using Marker analysis (Kyushu Pro Search LLP, Fukuoka, Japan). All peak areas were aligned into one datasheet, and the errors of peak intensities were corrected using internal standards. Noise peaks were deleted after comparison with the peaks detected in blank samples. The metabolites were identified by comparing the retention times and m/z values with a standard dataset provided by Kyushu Pro Search LLP.

### Quantitative reverse transcription polymerase chain reaction

Total RNA was extracted from the liver and muscle tissue with TRIzol reagent (Invitrogen, Carlsbad, CA), and cDNA was synthesized with PrimeScript RT Master Mix (Takara Bio, Tokyo, Japan). Real-time polymerase chain reaction (PCR) was performed using TB Green Premix Ex Taq II (Takara Bio, Tokyo, Japan). The primer sequences used in this study are listed in Additional file 6: Table S1.

### Statistical analysis

Data were analyzed using JMP Pro Version 16 (SAS Institute Inc., Cary, NC, USA). Continuous data were expressed as the mean value and standard deviation (SD) or standard error of the mean (SE). The difference between means was analyzed using Student’s t-test. Values of *P* < 0.05 were considered statistically significant.

## Results

### Dexamethasone induced hyperammonemia in Otc^spf−ash^ mice

**Fig. 1:**
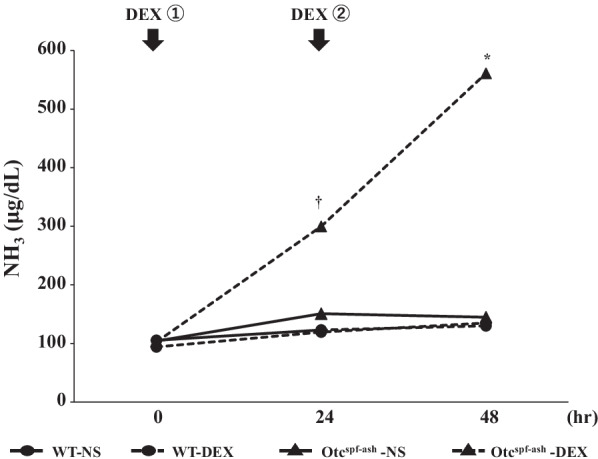
The time-course of serum ammonia levels. The blood samples were collected at 0, 24, and 48 h after the first DEX administration. Ammonia levels at 24 h after DEX administration were increased in the Otc^spf−ash^-DEX group (*P* < 0.05; vs. dex-matched controls, *P* = 0.06; vs. genotype-matched controls). Ammonia levels at 48 h after DEX administration were further increased in the Otc^spf−ash^-DEX group (*P* = 0.06; vs. dex-matched controls, *P* < 0.05; vs. genotype-matched controls). Data are expressed as the mean ± SD. WT-NS and WT-DEX, n = 3/group; Otc^spf−ash^- NS and Otc^spf−ash^-DEX, n = 5/group. ^†^*P* < 0.05; vs. dex-matched controls, *P < 0.05; vs. genotype-matched controls. NS; normal saline, DEX; dexamethasone

To evaluate the effects of DEX administration (20 mg/kg/body) on ammonia metabolism, the serum ammonia levels of the mice were measured at 0, 24, and 48 h after DEX administration. The ammonia levels in Otc^spf−ash^ mice were similar to those of WT mice at 0 h (103.2 ± 8.3 and 99.6 ± 10.8 μg/dL, *P* = 0.80; Fig. 1). The ammonia levels in Otc^spf−ash^ mice that were administered DEX were rapidly elevated at 24 h (WT-normal saline (NS) 123.3 ± 10.8 μg/dL vs. WT-DEX 119.3 ± 31.1 μg/dL, *P* = 0.86, WT-DEX 119.3 ± 31.1 μg/dL vs. Otc^spf−ash^-DEX 299.8 ± 130.6 μg/dL, *P* < 0.05, Otc^spf−ash^-NS 150.8 ± 25.4 μg/dL vs. Otc^spf−ash^-DEX 299.8 ± 130.6 μg/dL, *P* = 0.06; Fig. 1). Further elevations in the ammonia levels in Otc^spf−ash^ mice that were administered DEX were observed at 48 h (WT-NS 130.0 ± 12.5 μg/dL vs. WT-DEX 135.0 ± 21.7 μg/dL, *P* = 0.75, WT-DEX 135.0 ± 21.7 μg/dL vs. Otc^spf−ash^-DEX 561.0 ± 357.7 μg/dL, *P* = 0.06, Otc^spf−ash^-NS 144.8 ± 35.6 μg/dL vs. Otc^spf−ash^-DEX 561.0 ± 357.7 μg/dL, *P* < 0.05; Fig. 1).

### Metabolomic analysis and the association with urea-cycle-related gene expression

**Fig. 2:**
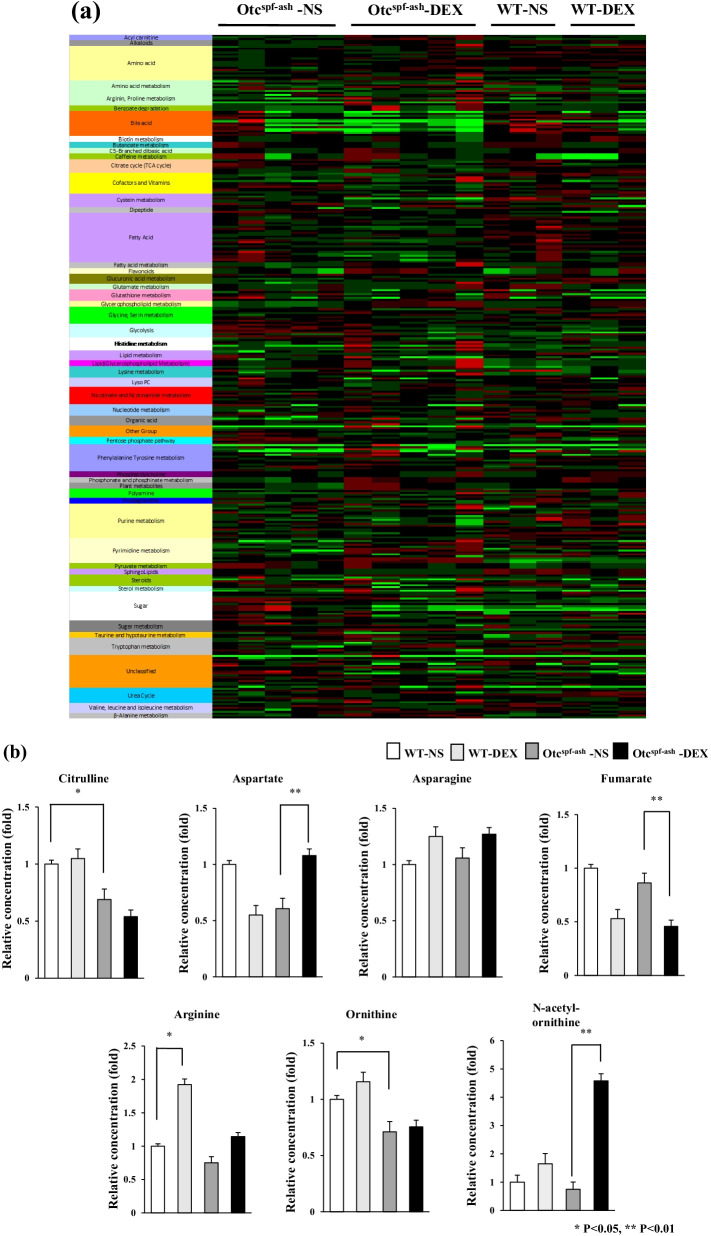
The levels of hepatic metabolites from WT and Otc^spf−ash^ mice that were administered DEX or NS. **a** Heat map analysis of metabolomics. It was generated by coloring the values of all data across their respective ranges. The color red indicates that the relative content of metabolites is high, while green indicates that they are low. The brightness of each color corresponds to the magnitude of the difference between the observed value and the average value. **b** The amounts of urea-cycle-related metabolites in WT and Otc^spf−ash^ mice, normalized to those in WT-NS were presented as mean ± SD. ∗ *p* < 0.05 and ∗ ∗*p* < 0.01. ASS1, arginosuccinate synthase 1; ASL, arginosuccinate lyase; ARG1, arginase 1; ORNT1, mitochondrial ornithine transporter 1; OTC, ornithine transcarbamylase; CPS1, carbamoyl-phosphate synthase 1. NS; normal saline, DEX; dexamethasone

We analyzed the levels of the metabolites extracted from the livers of the mice (Fig. 2a, b). The heat maps of metabolites other than the urea-cycle-related metabolites showed no significant changes (Fig. 2a). OTC deficiency resulted in a decrease in citrulline and ornithine in comparison to the Otc^spf−ash^-NS mice and the WT-NS mice (*P* < 0.05, Fig. 2b). The levels of citrulline, ornithine, and arginine did not differ significantly between Otc^spf−ash^-DEX and Otc^spf−ash^-NS. The levels of citrulline and ornithine did not differ significantly between WT-DEX and WT-NS, whereas DEX administration increased arginine in WT mice. DEX administration resulted in a decrease in fumarate and an increase in N-acetyl ornithine in Otc^spf−ash^ mice. DEX administration also increased aspartate in Otc^spf−ash^ mice but decreased aspartate in the WT mice. Glutamine tended to increase in WT mice by DEX administration (*P* = 0.12), although L-glutamine did not increase in Otc^spf−ash^ mice by DEX administration (Additional file 3: Fig. S2a).

### Quantitative PCR analysis of urea-cycle-related genes

**Fig. 3:**
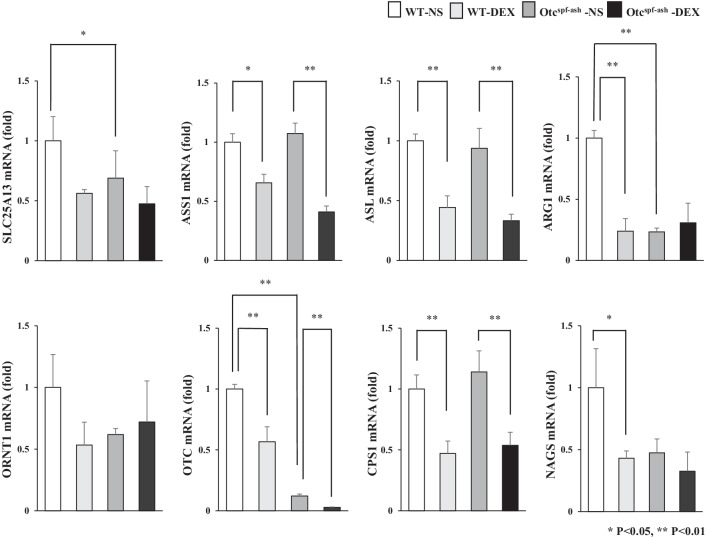
The urea-cycle-related gene expression levels in WT and Otc^spf−ash^ mice administered DEX or NS. Quantitative RT-PCR analysis of the urea-cycle-related genes. The gene expression levels normalized to those of WT-NS and were presented as mean ± SE. ∗*p* < 0.05 and ∗∗*p* < 0.01. SLC25A13, Solute Carrier Family 25 Member 13; ASS1, arginosuccinate synthase 1; ASL, arginosuccinate lyase; ARG1, arginase 1; ORNT1, mitochondrial ornithine transporter 1; OTC, ornithine transcarbamylase; CPS1, carbamoyl-phosphate synthase 1; NAGS, N-acetylglutamate synthetase. NS; normal saline, DEX; dexamethasone

We examined urea-cycle-related gene expression levels of the WT and Otc^spf−ash^ livers (Fig. 3), since it was considered that the cause of the increase in aspartate and the decrease in fumarate may be the change in urea-cycle-related gene expression. OTC deficiency significantly decreased the gene expressions of Solute Carrier Family 25 Member 13 (*SLC25A13), ARG1,* ornithine transcarbamylase (*OTC*), and in Otc^spf−ash^-NS mice compared to WT-NS mice. DEX administration significantly decreased the gene expressions of arginosuccinate synthase 1 (*ASS1*), and arginosuccinate lyase (*ASL*), *CPS1*, *OTC* in both WT and Otc^spf−ash^ mice. DEX administration significantly decreased *ARG1* and N-acetylglutamate synthetase (*NAGS)* gene expression in WT mice but not in Otc^spf−ash^ mice and did not affect mitochondrial ornithine transporter 1 (*ORNT1*) expression in either WT or Otc^spf−ash^ mice. The mRNA expression of glutamine synthetase (*GS*) was not increased in Otc^spf−ash^ and WT mice after the administration of DEX (Additional file 3: Fig. S2b).

### Quantitative PCR analysis of genes related with catabolism and anabolism

**Fig. 4:**
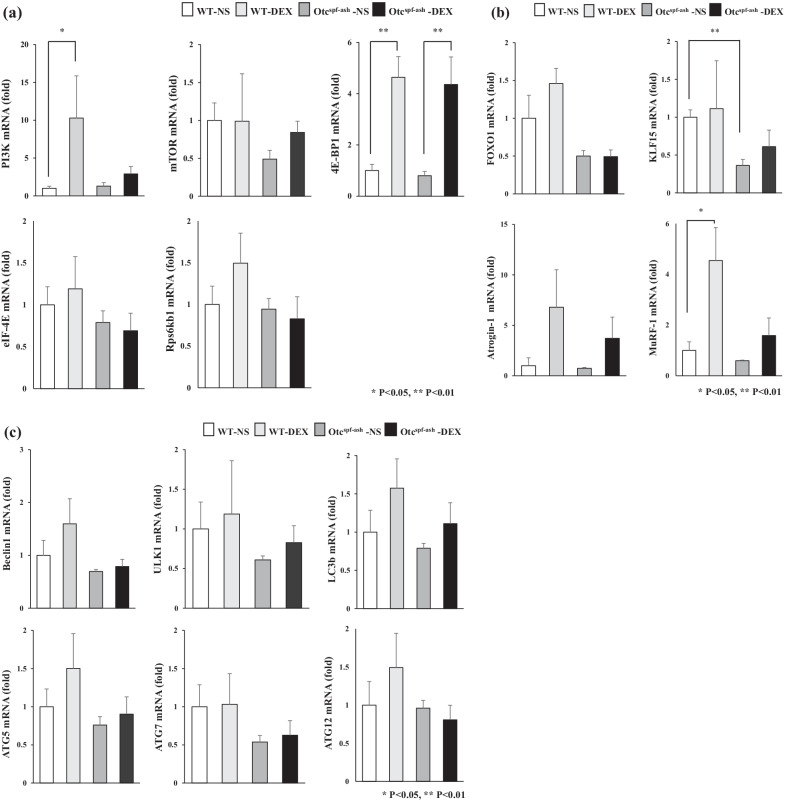
The gene expression levels related with catabolism and anabolism in WT and Otc^spf−ash^ muscles administered DEX or NS. **a** Quantitative RT-PCR analysis of the anabolism-related genes. **b** Quantitative RT-PCR analysis of the catabolism-related genes. **c** Quantitative RT-PCR analysis of the autophagy-related genes. The gene expression levels normalized to those of WT-NS and were presented as mean ± SE. ∗*p* < 0.05 and ∗ ∗*p* < 0.01. PI3K, phosphatidylinositol-3 kinase; mTOR, mammalian target of rapamycin; 4E-BP1, Eukaryotic translation initiation factor 4E-binding protein 1; eIF4E, eukaryotic translation initiation factor 4E; Rps6kb1, Ribosomal Protein S6 Kinase B1; FOXO1, forkhead box protein O1; KLF15, Kruppel Like Factor 15; MuRF-1, Muscle RING-finger protein-1; ULK1, Unc-51 Like Autophagy Activating Kinase 1; ATG, Autophagy related. NS; normal saline, DEX; dexamethasone

We examined gene expression levels related with anabolism and catabolism in WT and Otc^spf−ash^ gastrocnemius muscles (Fig. 4). The gene expression levels related with anabolism, including phosphatidylinositol-3 kinase (*PI3K),* Eukaryotic translation initiation factor 4E-binding protein 1 *(4E-BP1)*, significantly increased in WT mice by DEX administration (Fig. 4a). The gene expression level of *4E-BP1* also increased in Otc^spf−ash^ mice. The gene expression levels of mammalian target of rapamycin *(mTOR)*, eukaryotic translation initiation factor 4E *(eIF-4E)*, Ribosomal Protein S6 Kinase B1 *(Rps6kb1)* did not change significantly in WT and Otc^spf−ash^ mice by DEX administration. The gene expression level of Muscle RING-finger protein-1 *(MuRF-1)* which is related with catabolism significantly increased in WT mice and tended to increase in Otc^spf−ash^ mice by DEX administration (Fig. 4b). OTC deficiency significantly decreased the gene expressions of Kruppel Like Factor 15 *(KLF15)* in Otc^spf−ash^-NS mice compared to WT-NS mice. The gene expression level of *Atrogin-1* tended to increase in the WT and Otc^spf−ash^ mice by DEX administration. The gene expression levels related with autophagy did not change in both WT and Otc^spf−ash^ mice by DEX administration (Fig. 4c). We also examined gene expression levels related with anabolism and catabolism in the WT and Otc^spf−ash^ livers and we found that the gene expression levels related with anabolism and catabolism did not significantly increase in WT and Otc^spf−ash^ livers (Additional file 4: Fig. S3).

### Histopathological change by dexamethasone administration in the liver and muscle

We evaluated the histopathological changes of the livers and muscles by DEX administration in WT and Otc^spf−ash^ mice. Lipid droplets appeared in the WT and Otc^spf−ash^ livers by DEX administration, but other histopathological changes were not newly appeared (Additional file 5: Fig S4a). Muscle atrophy was not observed by DEX administration in WT and Otc^spf−ash^ mice on HE staining (Additional file 5: Fig S4b).

## Discussion

OTCD is caused by the loss of function in the OTC, which is responsible for ureagenesis. It is characterized by hyperammonemia, which leads to a brain injury or a fatal outcome [14]. Recent studies on OTCD revealed a broad spectrum of genetic defects resulting in diverse phenotypes [15]. Since individuals with mild OTCD can lead a normal life until severe environmental stress triggers a hyperammonemic crisis, late-onset presentations of UCDs often go unrecognized and may be life-threatening [16, 17]. Understanding the mechanism of hyperammonemia in patients with OTCD who received corticosteroids is important for a better treatment strategy.

Corticosteroid-induced hyperammonemia in OTCD patients is thought to be associated with skeletal muscle catabolism [7]. Corticosteroid-induced myopathy is a toxic noninflammatory myopathy caused by corticosteroid administration. Corticosteroid-induced myopathy typically develops with doses higher than 10 mg prednisone equivalents/day administered for at least 4 weeks [18, 19]. In the mouse model of corticosteroid-induced muscle atrophy, the ratio of muscle weight to body weight significantly declined 18 days after DEX administration [20]. At the molecular level, high doses of prednisolone for 3 days lead to an increase in protein catabolism in human skeletal muscle and amino acids in the arterial blood [21]. In this study, muscle atrophy was not observed on HE staining, but the gene expression levels related with catabolism were increased. It was suggested that corticosteroid-induced muscle catabolism could be involved in hyperammonemia.

Corticosteroid-induced hyperammonemic encephalopathy has been reported in 11 adult-onset OTCD patients, including two cases presented in this study (Table 1) [6–8, 22–26]. The time to onset varies from 2 to 56 days, which could be due to the residual enzymatic activity or the administered dose of corticosteroids. The mean ammonia levels in the OTCD patients who were administered corticosteroids was 1066 μg/dL (Table 1), which was significantly higher than the change in serum ammonia levels in OTC patients with infectious diseases that promote catabolism (172 μmol/L ≒ 293 μg/dL) [27]. The changes in serum ammonia levels from baseline did not differ significantly between infectious and dietary precipitants (172 vs. 147 μmol/L (≒ 293 vs. 254 μg/dL)) [27]. Thus, elevated ammonia levels in patients with OTCD who received corticosteroids were not thought to be solely due to corticosteroid-induced catabolism.

**Table 1:** Case reports of adult-onset OTC deficiency induced by corticosteroids

n	Ref	Age	M/F	Primary disease	Steroid	Max NH_3_ (µg/dL)	Symptom	Onset (days)	Treatment	Outcome
1	[6]	67	M	NSIP	Predonisone	4257	Seizure	–	–	Dead
2	[7]	56	M	Glottic edema	Unknown	320	Coma	4	HD	Alive
3	[8]	24	M	Deviated septum	Dexamethasone 8 mg/day	885	Vagueness	2	–	Dead
4	[8]	39	M	Knee arthritis	Cortisone	1124	Headache, nausea	2	–	Dead
5	[22]	45	M	Knee arthritis	Cortisone	700	Coma	9	–	Dead
6	[23]	36	M	Hearing defect	Predonisone 60 mg/day	1185	Coma	14	P + B	Alive
7	[24]	26	F	Preterm labor	Betamethasone	507	Coma	4	B + CHDF	Alive
8	[25]	58	F	Asthma	Methylprednisolone	477	Coma	5	P + B + HD	Alive
9	[26]	19	M	Leukoplakia	Betamethasone 1 mg/day	> 500	Coma	56	–	Dead
10	This report	45	M	Meniere’s disease	Predonisone 60 mg/day	784	Disorientation	5	HF-CHDF	Alive
11	This report	30	M	Asthma	Predonisone 30 mg/day	423	Disorientation	7	CHDF	Alive

*M* male, *F* female, *NSIP* nonspecific interstitial pneumonia, *P* phenylbutyrate, *B* benzoate, *HD* hemodialysis, *HF-CHDF* high-flow continuous hemodiafiltration

We examined urea-cycle-related gene expression levels of the WT and Otc^spf−ash^ livers because it was considered that the increase in aspartate and the decrease in fumarate may be caused by the altered urea-cycle-related gene expression. DEX administration significantly decreased the gene expressions of *ASS1*, *ASL*, *OTC*, and *CPS1* in both WT and Otc^spf−ash^ mice, and this was considered to be an important cause of the exacerbation of ammonia levels. These results indicate that corticosteroid administration induced hyperammonemia in Otc^spf−ash^ mice by not only muscle catabolism but also suppressing urea-cycle-related gene expressions. Although the urea-cycle-related gene expression changes by corticosteroid administration in this study were different from the previous reports in vitro [10–12], the different experimental conditions could have affected the results. Suppressed *OTC* gene expression might induce increased ornithine concentration in the OTCD patients and the increased ornitine might suppress *ARG1* gene expression. Ornithine might be rapidly converted to the other metabolites and we could not detect the metabolomic alternation in Otc^spf−ash^ mice liver.

In the patient in case 1, we identified an R40H (c.119G > A) mutation in the *OTC* gene that is associated with late-onset OTCD, and such patients were born within a limited area of the Kyushu Island in Southern Japan [28], which is the area where the current case was detected. Nishiyori et al. reported that the residual enzyme activity of R40H OTC accounted for 28% of the activity of controls [28]. Although the outcome can be fatal if not properly managed, this mutation is usually associated with a mild phenotype [29, 30]. An R40H mutation in the *OTC* gene was identified in case 1; however, the hyperammonemic encephalopathy was rapidly exacerbated by corticosteroid administration, and 5 days of hemodialysis was required to normalize serum ammonia levels. The rapid exacerbation of hyperammonemia could be associated with the suppression of urea-cycle-related gene expressions by corticosteroids.

Although we focused on the ammonium homeostasis in the liver and muscle in this study, ammonia is produced by not only liver and skeletal muscle, but also intestines and kidneys [31]. The small intestine produces ammonia through the catabolism of glutamine by glutaminase [32], and the large intestine produces ammonia by bacterial deaminase and bacterial urease [33]. Glucocorticoids may increase ammonia in the small intestine because glucocorticoids upregulate glutaminase gene expression in human intestinal epithelial cells [34]. The kidney produces free ammonium ions that are either excreted into the urine or released into the systemic circulation [35]. Glucocorticoids may have a positive effect in terms of ammonia detoxification because glucocorticoids increase the fractional excretion of urea in rat kidneys [36].

We saved two adult-onset OTCD patients by multimodal treatments including dialysis and they recovered completely from severe hyperammonemia (Additional file 1 and Additional file 2: Fig. S1). The treatment goal of UCD-related hyperammonemia is to reduce the serum ammonia level as quickly as possible because the highest ammonia blood concentration at onset (ammonia > 600 μg/dL) is associated with poor prognosis and serious neurological sequelae [37]. The treatment regimen includes hemodialysis, provision of a high-calorie and no-protein diet (to prevent further catabolism), and the administration of L‐arginine and ammonia-scavenging medications (sodium phenylacetate, sodium benzoate). Since OTC is not saturated with ornithine in Otc^spf−ash^ mice, the administration of the urea cycle intermediate amino acids enhances the OTC reaction, and the ammonia metabolism of Otc^spf−ash^ mice is partially normalized [38]. The intermediate amino acids of the urea cycle, such as arginine, are important to avoid neurological symptoms in the long-term treatment of UCD patients. However, these might not be effective because of the suppression of urea-cycle-related gene expressions by corticosteroid administration in acute UCD decompensations. An immediate application of blood-purifying treatment should be considered to prevent death and serious neurological sequelae because benzoate is known to be insufficient for hyperammonemic comas (ammonia > 250 μmol/L, i.e., 425 μg/dL), even when combined with phenylacetate [39]. When plasma ammonia levels exceed 200 µmol/L (≒ 340 μg/dL), renal replacement therapy is recommended [9]. Since urea-cycle-related gene expressions were suppressed in OTCD with corticosteroids, we need to consider the early intervention of renal replacement therapy in the cases of OTCD patients treated with corticosteroids. Among patients with OTCD who received corticosteroids, five who were treated by means of hemodialysis or continuous hemodiafiltration were recovered, while five out of the six who did not receive any blood-purifying treatment died (Table 1). To reduce ammonia levels more rapidly, we might also consider high-volume filtrate hemodiafiltration (high-flow continuous hemodiafiltration or online hemodiafiltration), which is proven to be effective in helping patients with fulminant hepatitis suffering from hepatic encephalopathy to recover their consciousness [40]. Corticosteroid-induced hyperammonemia was observed in not only patients with OTCD but also in patients with UCD [9]. Since it is presumed that there is a high possibility for urea-cycle-related gene expressions to be suppressed in UCD patients, we recommend early intervention by means of renal replacement therapy for hyperammonemic patients who are likely to have UCDs and are treated with corticosteroids.

## Conclusions

We elucidated that corticosteroid administration decreased urea-cycle-related gene expressions in both WT and Otc^spf−ash^ mice. Since the urea cycle function is natively impaired in Otc^spf−ash^ mice, it is reasonable for corticosteroid administration to result in the rapid development of severe hyperammonemia. This result might explain why hyperammonemia induced by corticosteroids in patients with OTCD tends to be more severe than that induced by other exacerbating factors such as inadequate diets and infections, which only increased catabolism. Given that renal replacement therapy is recommended for severe hyperammonemia with serum ammonium levels exceeding 340 μg/dL, we should not hesitate to engage in early interventions by means of renal replacement therapy to combat corticosteroid-induced hyperammonemia in patients with UCD to avoid brain injuries or fatal outcomes.

## Supplementary Information

**Additional file 1**. Case series of the two late-onset OTCD patients who received corticosteroids.

**Additional file 2**. The clinical courses of case 1 and case 2.

**Additional file 3**. The changes related with glutamine metabolism in the livers of Otc^spf-ash^ and WT mice that were administered DEX or NS.

**Additional file 4**. The gene expression levels related with catabolism and anabolism in WT and Otc^spf-ash^ livers administered DEX or NS.

**Additional file 5**. Histology of the liver and gastrocnemius muscle from WT and Otcspf-ash mice administered DEX or NS.

**Additional file 6**. The sequences of primers used in the present study.

## Data Availability

The data used to support the findings of this study are included within the article.

## Abbreviations

ARG1: Arginase 1
ASL: Arginosuccinate lyase
ASS1: Arginosuccinate synthase 1
CPS1: Carbamoyl-phosphate synthase 1
DEX: Dexamethasone
eIF-4E: Eukaryotic translation initiation factor 4E
4E-BP1: 4E-binding protein 1
GAPDH: Glyceraldehyde 3-phosphate dehydrogenase
GS: Glutamine synthetase
KLF15: Kruppel Like Factor 15
mTOR: Mammalian target of rapamycin
MuRF-1: Muscle RING-finger protein-1
NAGS: N-acetylglutamate synthetase
NS: Normal saline
ORNT1: Mitochondrial ornithine transporter 1
OTC: Ornithine transcarbamylase
OTCD: Ornithine transcarbamylase deficiency
PI3K: Phosphatidylinositol-3 kinase
Rps6kb1: Ribosomal Protein S6 Kinase B1
SD: Standard deviation
SE: Standard error of the mean
SLC25A13: Solute Carrier Family 25 Member 13
UCDs: Urea cycle disorders
WT: Wild-type
