# Supplementary material for: Corticosteroid suppresses urea-cycle-related gene expressions in ornithine transcarbamylase deficiency
Source: BMC Gastroenterol. 2022 Mar 28;22:144. doi: 10.1186/s12876-022-02213-0 (PMC8962007; doi:10.1186/s12876-022-02213-0)
Supplement: Supplementary file 1 — Additional file 1. Case series of the two late-onset OTCD patients who received corticosteroids. [file 12876_2022_2213_MOESM1_ESM.docx]

Case series

The clinical courses and blood tests of the two late-onset OTCD patients who received corticosteroids are summarized in Fig. S1.

Case 1

A 44-year-old previously healthy Japanese man was admitted to a local hospital owing to hyperammonemia and disorientation after prednisolone treatment for sudden hearing loss. Although he recovered naturally, the cause was not clear. One year after this admission, he was admitted again to this hospital because of Meniere’s disease. There was neither a family history of metabolic disease, liver disease, nor evidence of alcohol use. Prednisolone was administered per os at a dose of 60 mg/day for Meniere’s disease. However, he suffered from disorientation, and his blood test revealed hyperammonemia (110 μg/dL) 5 days later. The following day, his consciousness level rapidly worsened, the serum ammonia level increased to 286 μg/dL, and he was transferred to our hospital for further evaluation and treatment. On arrival at our hospital, his serum ammonia concentration rose to 784 μg/dL, and a head CT scan revealed cerebral edema. Since his neurological deterioration resulted in a coma, we started mechanical ventilation and high-flow continuous hemodiafiltration combined with L-arginine, lactulose, and rifaximin administration. The serum ammonia level was reduced by these treatments and became normal 5 days later. His consciousness level also improved gradually, and he regained consciousness within the next few days. He was discharged from our hospital and returned to his previously active life while maintaining a low-protein diet. His repeated serum ammonia level was 50–60 μg/dL. As the drastic increase in serum ammonia is not typical of hepatic hyperammonemia, UCDs were indicated as the most likely cause of impaired consciousness. After obtaining the patient’s informed consent, we performed a genomic analysis of the OTC gene, and Arg40His (c.119G > A) in exon 2 of the OTC gene was identified.

Case 2

A 30-year-old previously healthy Japanese man was admitted because of disorientation to our hospital. His uncle and cousin died of OTCD, and another cousin was diagnosed with OTCD. There was neither a history of liver disease nor evidence of alcohol use. One week before admission, he began receiving 30 mg/day of oral prednisolone for bronchial asthma. On admission, his serum ammonia level was 423 μg/dL. We administered L-arginine at first. However, L-arginine treatment did not improve his consciousness level, and the CT scan revealed cerebral edema. We started continuous hemodiafiltration immediately to remove ammonia rapidly from his circulation. His serum ammonia level was normalized, and he regained consciousness 24 h later. He was discharged from our hospital with no neurological sequelae. A few months later, the condition of the patient was good during a follow-up visit, and his serum ammonia level was 30 μg/dL. He was diagnosed with OTC deficiency thanks to a combination of his history, clinical presentation, amino acid analysis, and orotic aciduria; however, the genomic analysis was not performed owing to lack of the agreement. The patient returned to his previously active life while maintaining a low-protein diet.
