# Supplementary material for: Corticosteroid suppresses urea-cycle-related gene expressions in ornithine transcarbamylase deficiency
Source: BMC Gastroenterol. 2022 Mar 28;22:144. doi: 10.1186/s12876-022-02213-0 (PMC8962007; doi:10.1186/s12876-022-02213-0)
Supplement: Supplementary file 2 — Additional file 2. The clinical courses of case 1 and case 2. [file 12876_2022_2213_MOESM2_ESM.pptx]

## Slide 1
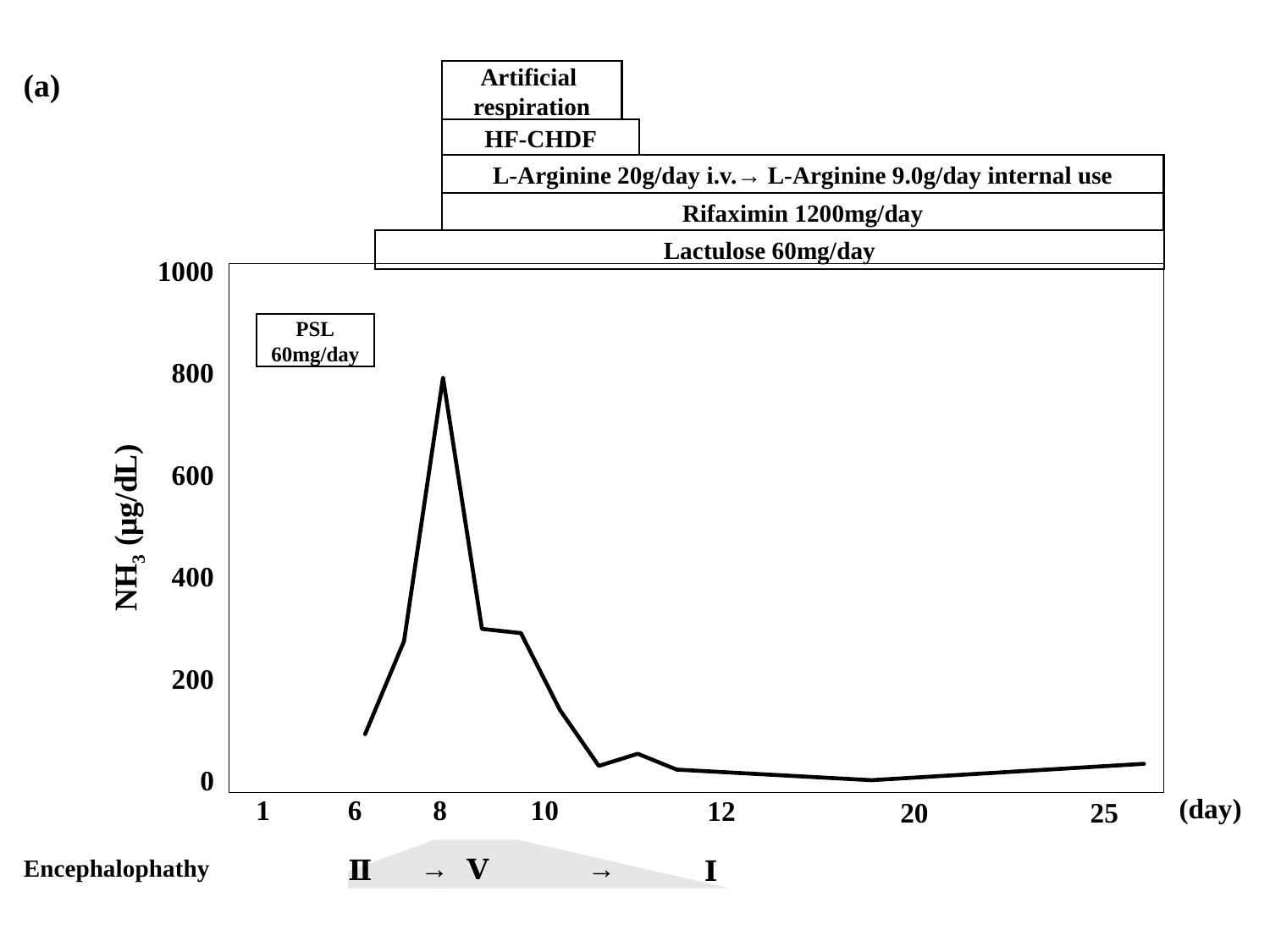

(a)
Artificial
respiration
HF-CHDF
L-Arginine 20g/day i.v.→ L-Arginine 9.0g/day internal use
Rifaximin 1200mg/day
Lactulose 60mg/day
1000
### Chart
| Category | |
|---|---|
| 42973 | None |
| 42976 | 110.0 |
| 42977 | 286.0 |
| 42978 | 784.0 |
| 42979 | 309.0 |
| 42980 | 301.0 |
| 42981 | 156.0 |
| 42982 | 50.0 |
| 42983 | 73.0 |
| 42984 | 43.0 |
| 42989 | 23.0 |
| 42996 | 54.0 |PSL
60mg/day
800
600
NH3 (μg/dL)
400
200
0
(day)
6
8
10
1
12
20
25
→
→
Ⅴ
Ⅱ
Encephalophathy
Ⅰ

## Slide 2
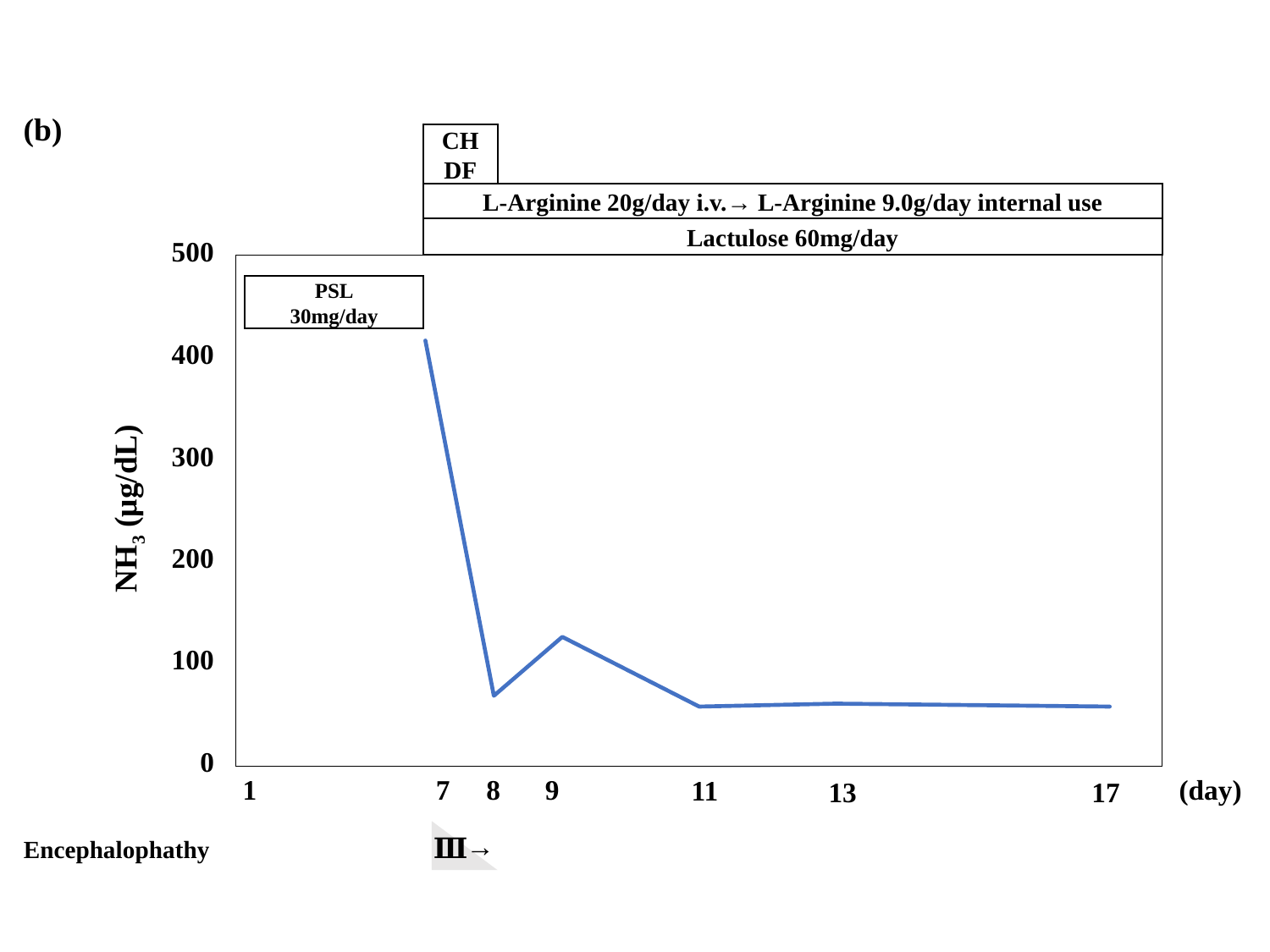

(b)
CHDF
L-Arginine 20g/day i.v.→ L-Arginine 9.0g/day internal use
Lactulose 60mg/day
500
### Chart
| Category | |
|---|---|
| 44164 | None |
| 44166 | 423.0 |
| 44167 | 61.0 |
| 44168 | 121.0 |
| 44170 | 50.0 |
| 44172 | 53.0 |
| 44176 | 50.0 |PSL
30mg/day
400
300
NH3 (μg/dL)
200
100
0
7
8
9
(day)
1
11
13
17
→
Ⅲ
Encephalophathy
