# Supplementary material for: Corticosteroid suppresses urea-cycle-related gene expressions in ornithine transcarbamylase deficiency
Source: BMC Gastroenterol. 2022 Mar 28;22:144. doi: 10.1186/s12876-022-02213-0 (PMC8962007; doi:10.1186/s12876-022-02213-0)
Supplement: Supplementary file 3 — Additional file 3. The changes related with glutamine metabolism in the livers of Otcspf-ash and WT mice that were administered DEX or NS. [file 12876_2022_2213_MOESM3_ESM.pptx]

## Slide 1
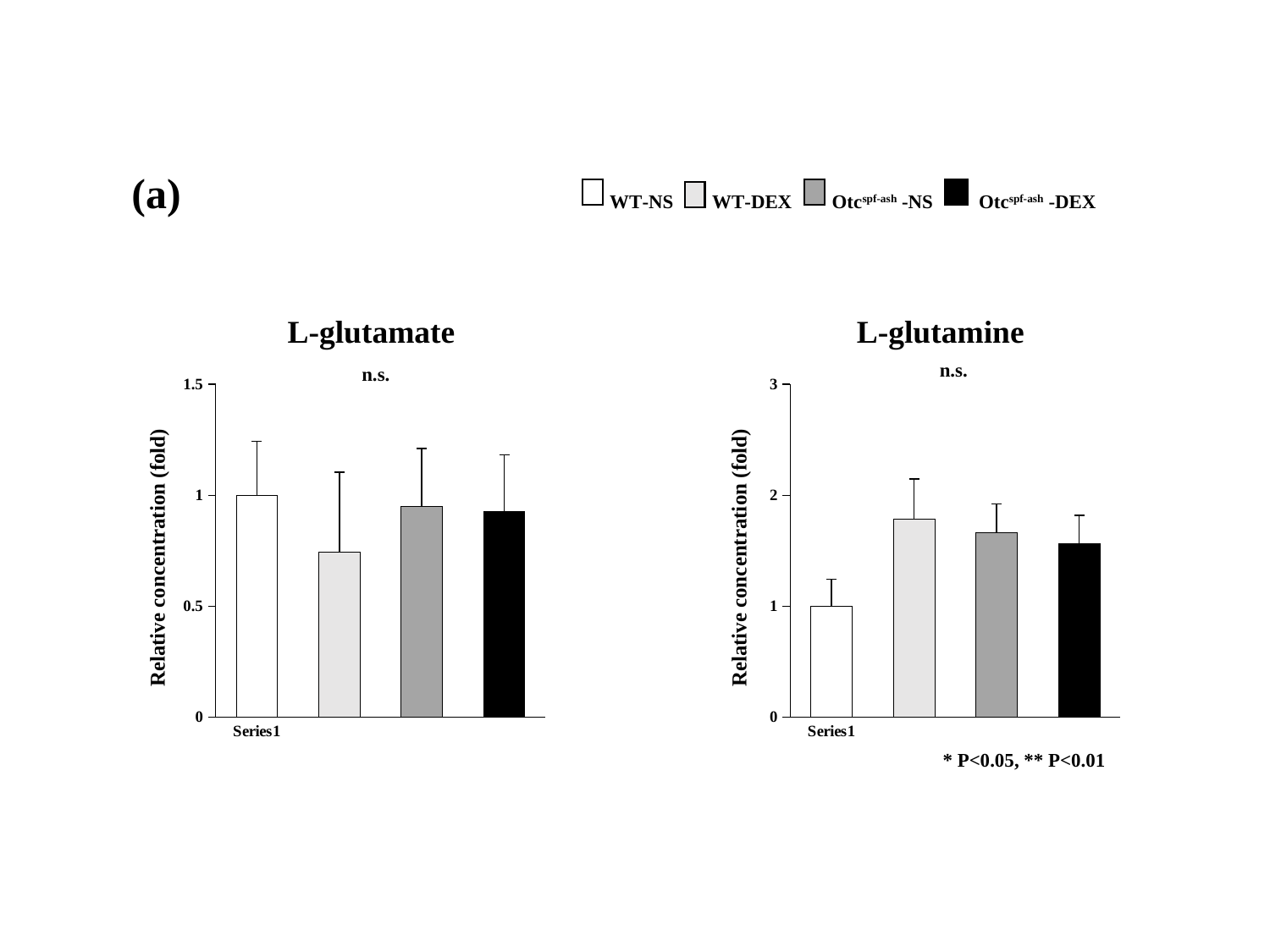

(a)
WT-NS
WT-DEX
Otcspf-ash -NS
Otcspf-ash -DEX
L-glutamate
L-glutamine
n.s.
n.s.
### Chart
| Category | 平均 |
|---|---|
| | 1.0000000000000002 |
| | 0.742321811871853 |
| | 0.9500651968946219 |
| | 0.928074785853277 |
### Chart
| Category | 平均 |
|---|---|
| | 1.0 |
| | 1.784338035039226 |
| | 1.6637860249583347 |
| | 1.5664363237266776 |Relative concentration (fold)
Relative concentration (fold)
* P<0.05, ** P<0.01

## Slide 2
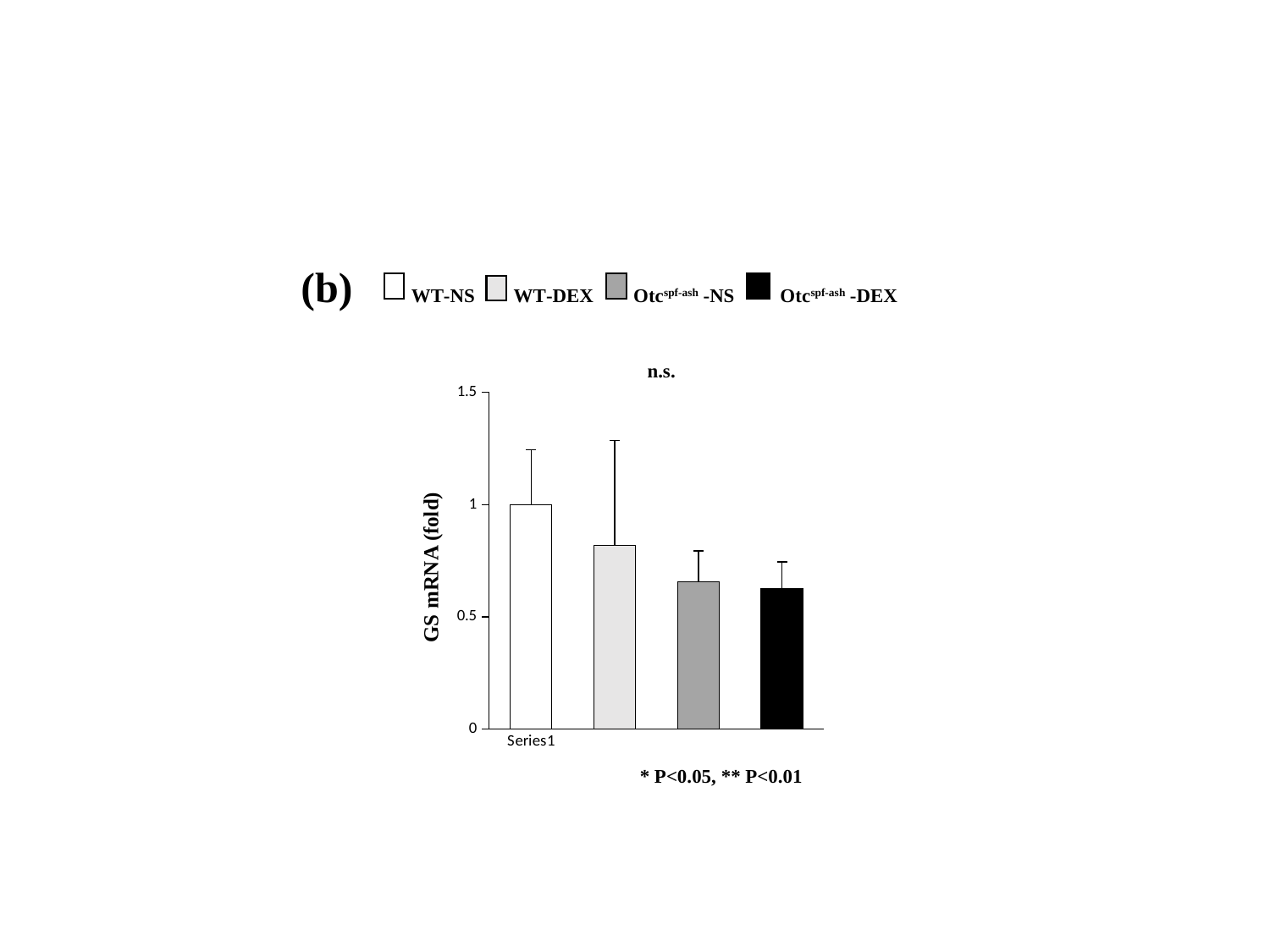

(b)
WT-NS
WT-DEX
Otcspf-ash -NS
Otcspf-ash -DEX
n.s.
### Chart
| Category | 平均 |
|---|---|
| | 1.0 |
| | 0.8191011141459065 |
| | 0.6554674353845868 |
| | 0.6276930542989976 |GS mRNA (fold)
* P<0.05, ** P<0.01
