# Supplementary material for: Corticosteroid suppresses urea-cycle-related gene expressions in ornithine transcarbamylase deficiency
Source: BMC Gastroenterol. 2022 Mar 28;22:144. doi: 10.1186/s12876-022-02213-0 (PMC8962007; doi:10.1186/s12876-022-02213-0)
Supplement: Supplementary file 4 — Additional file 4. The gene expression levels related with catabolism and anabolism in WT and Otcspf-ash livers administered DEX or NS. [file 12876_2022_2213_MOESM4_ESM.pptx]

## Slide 1
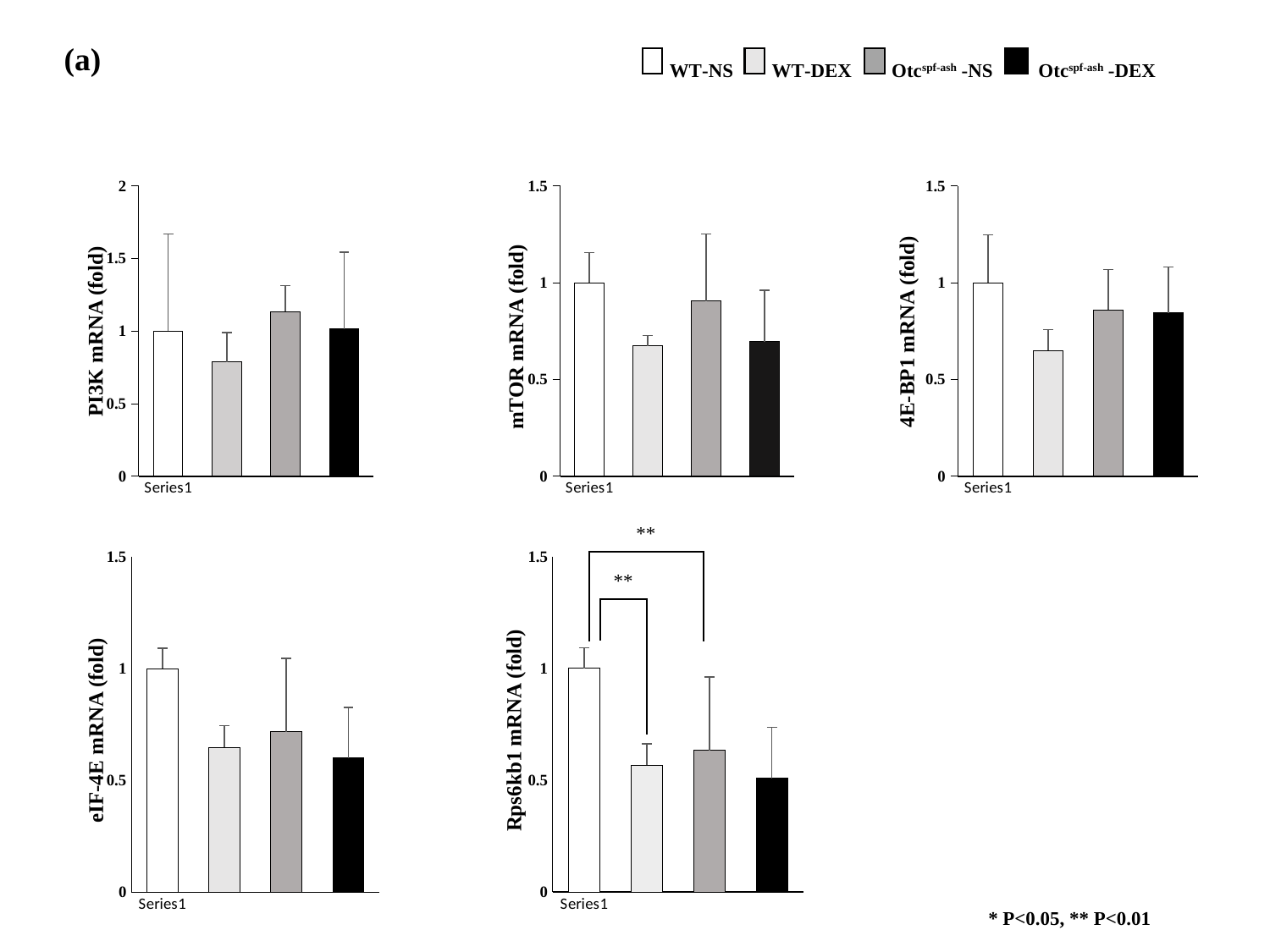

(a)
WT-NS
WT-DEX
Otcspf-ash -NS
Otcspf-ash -DEX
### Chart
| Category | 平均 |
|---|---|
| | 1.0 |
| | 0.7872381096618598 |
| | 1.1342893431120642 |
| | 1.014701585390002 |
### Chart
| Category | 平均 |
|---|---|
| | 1.0000000000000002 |
| | 0.6728447936468956 |
| | 0.9052401694548162 |
| | 0.6959534627530486 |
### Chart
| Category | 平均 |
|---|---|
| | 1.0 |
| | 0.649248240279892 |
| | 0.8601961693714916 |
| | 0.843654902156399 |
### Chart
| Category |
|---|PI3K mRNA (fold)
4E-BP1 mRNA (fold)
mTOR mRNA (fold)
**
### Chart
| Category | 平均 |
|---|---|
| | 0.9999999999999997 |
| | 0.5663010535973632 |
| | 0.6331566342307221 |
| | 0.5086613940544575 |
### Chart
| Category | 平均 |
|---|---|
| | 1.0 |
| | 0.6477230380321434 |
| | 0.7181480154235187 |
| | 0.6007193925338903 |**
eIF-4E mRNA (fold)
Rps6kb1 mRNA (fold)
* P<0.05, ** P<0.01
**

## Slide 2
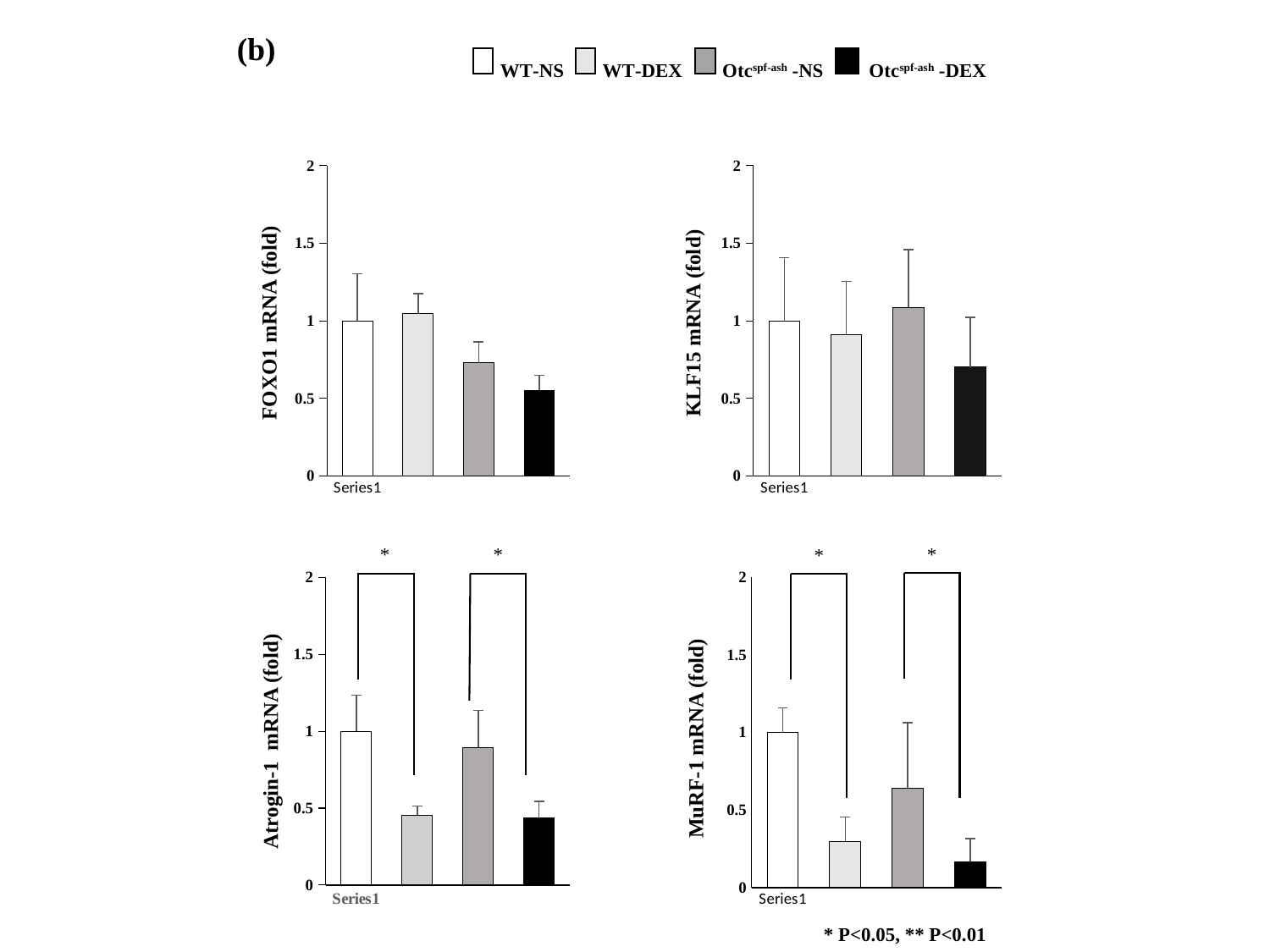

(b)
WT-NS
WT-DEX
Otcspf-ash -NS
Otcspf-ash -DEX
### Chart
| Category | 平均 |
|---|---|
| | 1.0 |
| | 0.9120002315200413 |
| | 1.0863391524561696 |
| | 0.7029716361816869 |
### Chart
| Category | 平均 |
|---|---|
| | 1.0 |
| | 1.0459972518844431 |
| | 0.7288570524590858 |
| | 0.5524388099073395 |FOXO1 mRNA (fold)
KLF15 mRNA (fold)
*
*
*
*
### Chart
| Category | 平均 |
|---|---|
| | 1.0 |
| | 0.2969415232613965 |
| | 0.6416974473183662 |
| | 0.16608979988585526 |
### Chart
| Category | 平均 |
|---|---|
| | 0.9999999999999999 |
| | 0.45444449802652603 |
| | 0.8934071288299924 |
| | 0.4355778524952708 |MuRF-1 mRNA (fold)
Atrogin-1 mRNA (fold)
* P<0.05, ** P<0.01

## Slide 3
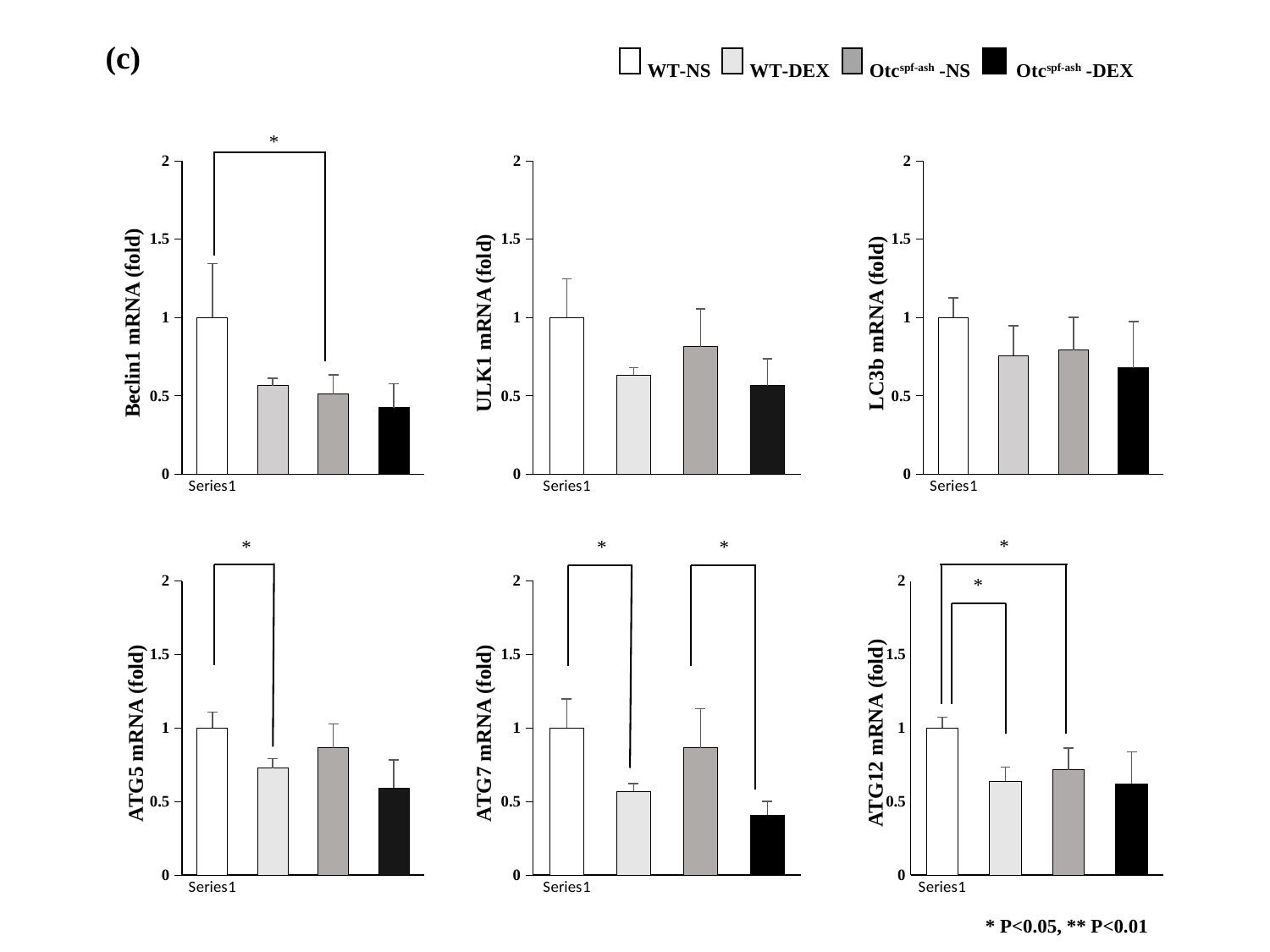

(c)
WT-NS
WT-DEX
Otcspf-ash -NS
Otcspf-ash -DEX
*
### Chart
| Category | 平均 |
|---|---|
| | 1.0000000000000002 |
| | 0.5650216462262394 |
| | 0.511325450568098 |
| | 0.42408931826954666 |
### Chart
| Category | 平均 |
|---|---|
| | 1.0000000000000002 |
| | 0.632324157986333 |
| | 0.8125508085020862 |
| | 0.5645551907694476 |
### Chart
| Category | 平均 |
|---|---|
| | 1.0000000000000002 |
| | 0.7577612509110571 |
| | 0.7911656922032856 |
| | 0.6774056340200951 |LC3b mRNA (fold)
ULK1 mRNA (fold)
Beclin1 mRNA (fold)
*
*
*
*
### Chart
| Category | 平均 |
|---|---|
| | 0.9999999999999999 |
| | 0.7275634075026437 |
| | 0.8664375261740875 |
| | 0.5913352760840689 |
### Chart
| Category | 平均 |
|---|---|
| | 1.0000000000000002 |
| | 0.5683590853877373 |
| | 0.8643167392049609 |
| | 0.4066324672836606 |
### Chart
| Category | 平均 |
|---|---|
| | 1.0 |
| | 0.6392230792691772 |
| | 0.7153161736307763 |
| | 0.6208935893665101 |*
ATG12 mRNA (fold)
ATG7 mRNA (fold)
ATG5 mRNA (fold)
* P<0.05, ** P<0.01
