# Supplementary figures and images for: Corticosteroid suppresses urea-cycle-related gene expressions in ornithine transcarbamylase deficiency
Source: BMC Gastroenterol. 2022 Mar 28;22:144. doi: 10.1186/s12876-022-02213-0 (PMC8962007; doi:10.1186/s12876-022-02213-0)

## Slide 1
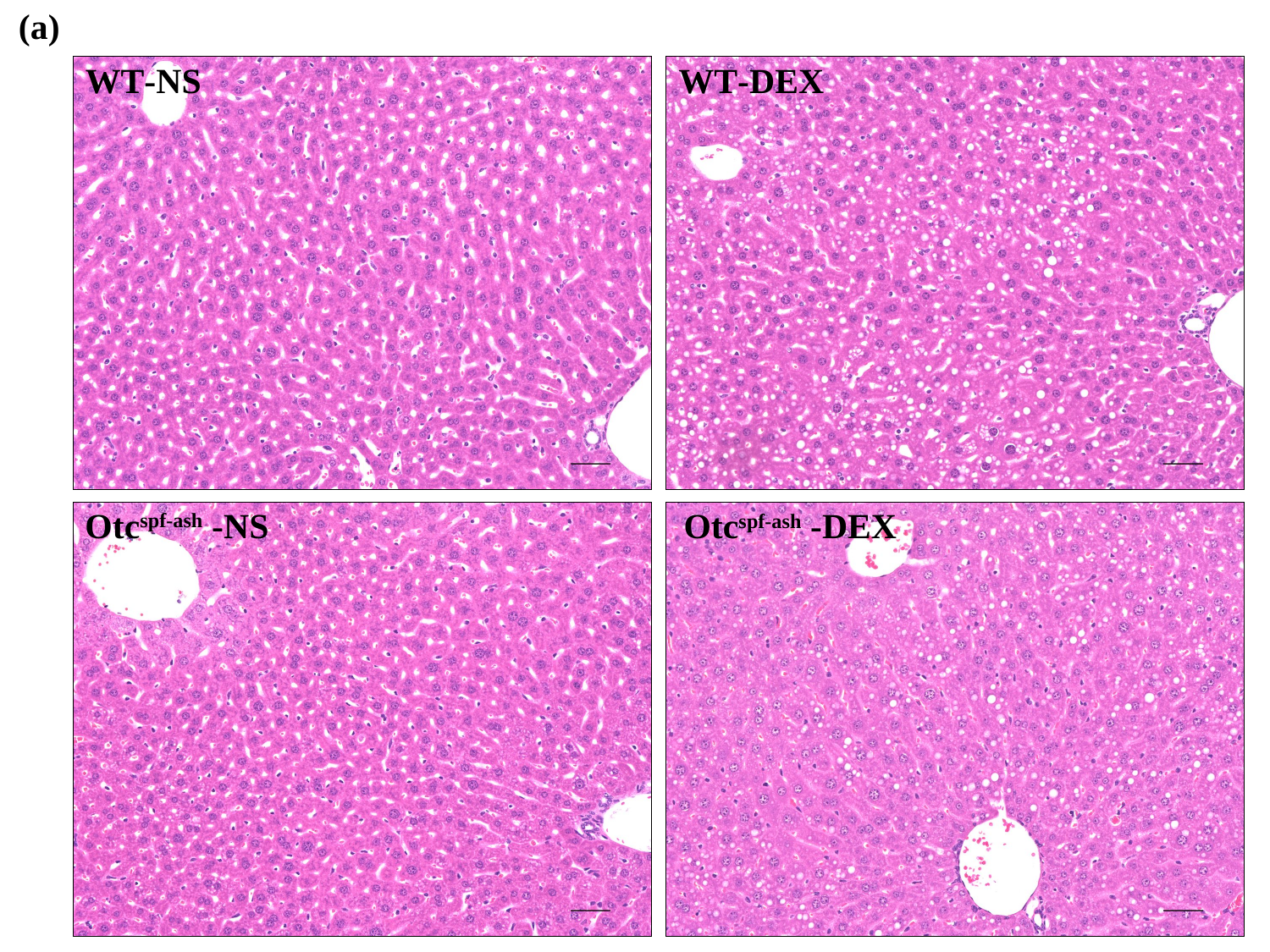

(a)
WT-DEX
WT-NS
Otcspf-ash -NS
Otcspf-ash -DEX

## Slide 2
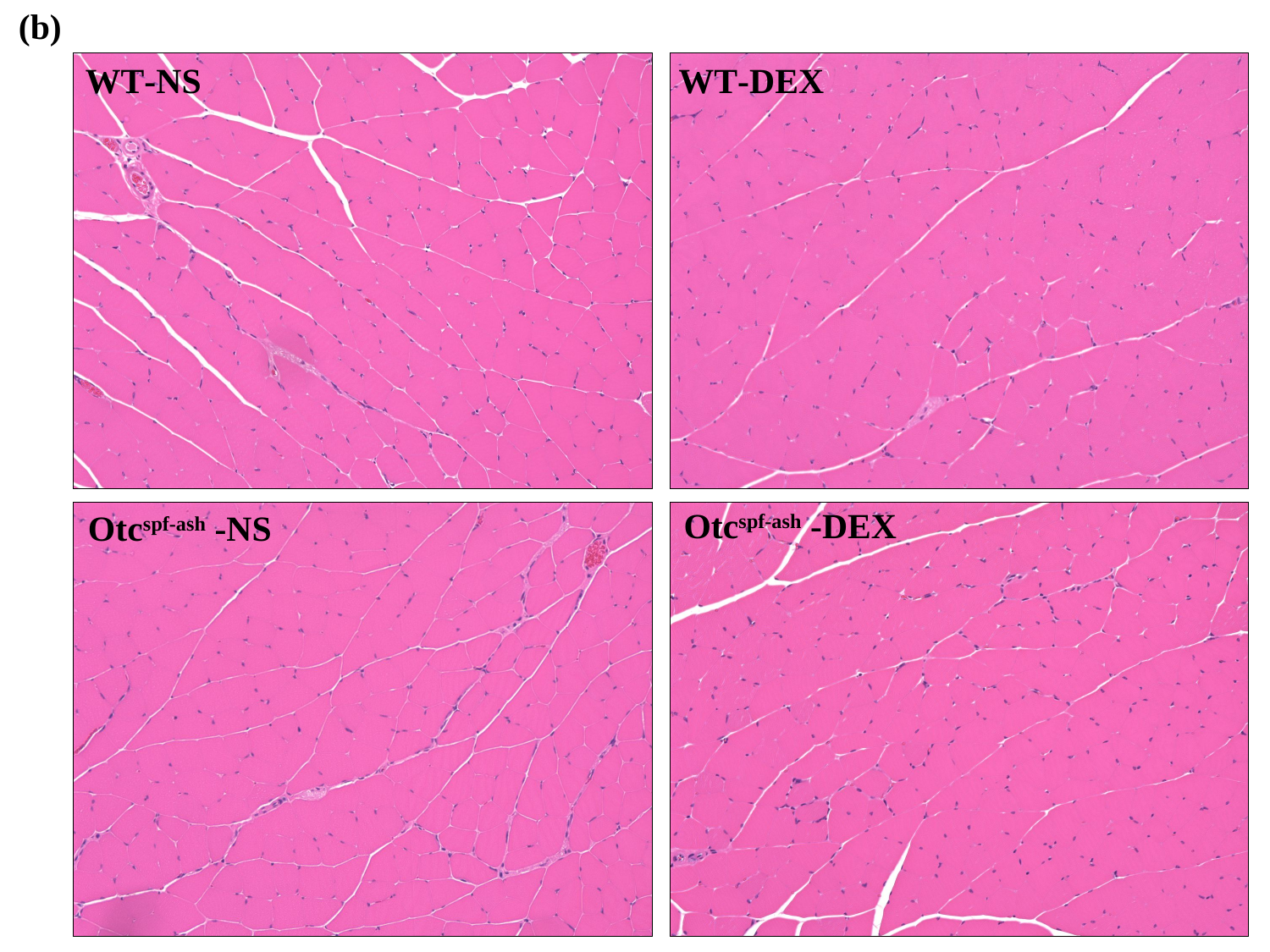

(b)
WT-DEX
WT-NS
Otcspf-ash -DEX
Otcspf-ash -NS

Supplement: Supplementary file 5 — Additional file 5. Histology of the liver and gastrocnemius muscle from WT and Otcspf-ash mice administered DEX or NS. [file 12876_2022_2213_MOESM5_ESM.pptx]

## Slide 1
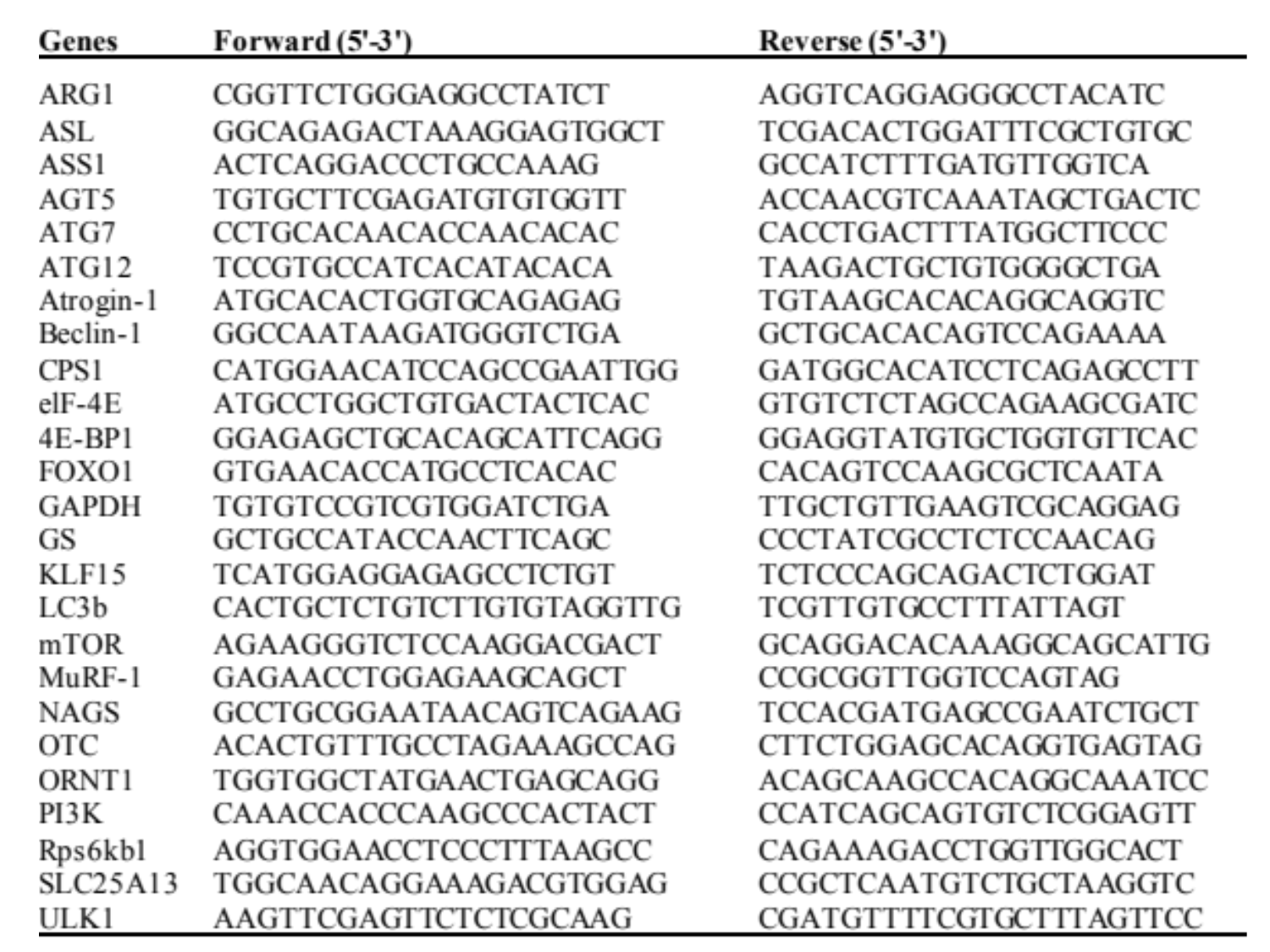

Supplement: Supplementary file 6 — Additional file 6. The sequences of primers used in the present study. [file 12876_2022_2213_MOESM6_ESM.pptx]
